# Do artificial sweeteners increase the risk of non-alcoholic fatty liver disease (NAFLD)?

**DOI:** 10.17179/excli2020-2745

**Published:** 2020-08-14

**Authors:** Tahany Abbas, Walaa Murad

**Affiliations:** 1 Histology Department, Faculty of Medicine, South Valley University, Qena 83523, Egypt

## ⁯

***Dear Editor,***

Recently, Emamat and colleagues published a review about NAFLD and a possible role of artificial sweeteners as a risk factor (Emamat et al., 2020[3]). NAFLD is the most frequent liver disorder in industrialized countries, which affects ~25 % of the population (Younossi et al., 2018[24]; Friedman et al., 2018[4]). In the past years, the prevalence of NAFLD increased in adults and in children and is also present in ~7 % of lean persons (Romero-Gómez et al., 2017[17]; Younossi et al., 2018[24]). Moreover, NAFLD represents a risk factor of primary liver cancer (AISF, 2017[1]; Trépo and Valenti, 2020[21]). In their review, Emamat et al. discuss the hypothesis that artificial sweeteners increase the risk of NAFLD (Emamat et al., 2020[3]). Artificial sweeteners or sugar substitutes are increasingly consumed to reduce caloric intake (Kakleas et al., 2020[14]; Suez et al., 2015[20]; Ruiz-Ojeda et al., 2019[18]; Uebanso et al., 2017[22]). The authors discuss the currently available evidence that artificial sweeteners alter the gut microbiota, which may increase the prevalence of NAFLD. 

Currently, much experimental effort is invested to gain a deeper understanding of liver disease (Jansen et al., 2017[13]; Godoy et al., 2013[8], 2015[9], 2016[10]; Ghallab et al., 2016[5], 2019[7][6]; Vartak et al., 2016[23]) and to identify compounds that cause an increased risk of hepatotoxicity (Grinberg et al., 2014[12], 2018[11]; Albrecht et al., 2019[2]; Kim et al., 2015[15]). Research in this field is often hampered by difficulties to extrapolate data from animal or *in vitro* experiments to the *in vivo* situation (Schenk et al., 2017[19]; Leist et al., 2017[16]). The review of Emamat et al. clearly shows that there is strong evidence that artificial sweeteners influence the composition of gut microbiota. However, further work including prospective and intervention studies are required to clarify if this mechanism really causes an increased risk of liver disease.

## Conflict of interest

The authors declare no conflict of interest.
